# Design of Experiments and Optimization of Monacolin K Green Extraction from Red Yeast Rice by Ultra-High-Performance Liquid Chromatography

**DOI:** 10.3390/foods13162509

**Published:** 2024-08-11

**Authors:** Lara Davani, Cristina Terenzi, Angela De Simone, Vincenzo Tumiatti, Vincenza Andrisano, Serena Montanari

**Affiliations:** 1 Department for Life Quality Studies, University of Bologna, Corso D’Augusto 237, 47921 Rimini, Italy; lara.davani2@unibo.it (L.D.); cristina.terenzi2@unibo.it (C.T.); vincenzo.tumiatti@unibo.it (V.T.); vincenza.andrisano@unibo.it (V.A.); 2 Department of Drug Science and Technology University of Torino, Via P. Giuria 9, 10125 Torino, Italy; angela.desimone@unito.it

**Keywords:** Monacolin K, DOE, UHPLC-DAD method, green extraction, enriched mouthwash

## Abstract

Monacolin K (MK), in red yeast rice (RYR) in the forms of lactone (LMK) and hydroxy acid (AMK), is known for its anti-hypercholesterolemic activity. Under the rising demand for natural bioactive molecules, we present a green ultrasound-assisted extraction (UAE) optimization study for MK in RYR. The development and validation of a fast, sensitive, selective, reproducible, and accurate ultra-high-performance liquid chromatography (UHPLC) method coupled to diode array detection for LMK and AMK allowed us to evaluate the MK recovery in different extract media. Firstly, the ethanol comparability to acetonitrile was assessed (recovery of 80.7 ± 0.1% for ethanol and 85.5 ± 0.2% for acetonitrile). Then, water/ethanol mixtures, with decreasing percentages of organic solvent, were tested by modulating temperature and extraction times. Water extractions at 80 °C for 10 min produced MK yield > 85%. Thus, UAE conditions were optimized by a DOE study using a water-based formulation (mouthwash). The optimal total MK extraction yield (86.6 ± 0.4%) was found under the following conditions: 80 °C, 45 min, 5 mg mL^−1^ (RYR powder/solvent). Therefore, the new single-process green approach allowed the simultaneous direct extraction of MK and mouthwash enrichment (MK concentration = 130.0 ± 0.6 µg mL^−1^), which might be tested for the prevention and treatment of periodontitis or oral candidiasis.

## 1. Introduction

Red yeast rice (RYR), obtained by fermenting white rice in the presence of fungal *Monascus* strains, has been used for centuries as food in Asian cuisine and as a preparation in traditional medicine [1,2].

Currently, RYR is considered the most effective food supplement in the prevention of vascular diseases and is used as the first nutraceutical approach in patients suffering from dyslipidaemia [1,2,3]. In 2022, the global red yeast rice market was valued at USD 387.97 million and will only increase [4]. 

Indeed, RYR contains monacolins, and Monacolin K (MK) is the most abundant active component. The increasing demand of RYR and the related MK is prompting research towards the fermentation optimization conditions by testing different *Monascus* strains, medium compositions, and types of solid matrixes (millet, corn, barley, wheat). This is to ensure a large-scale factory production and to foster market competitiveness [5,6]. 

Specifically, in RYR, MK can be found not only in the lactone form (LMK) but also in the hydrolyzed hydroxy acid form (AMK) [7].

**Figure 1:**
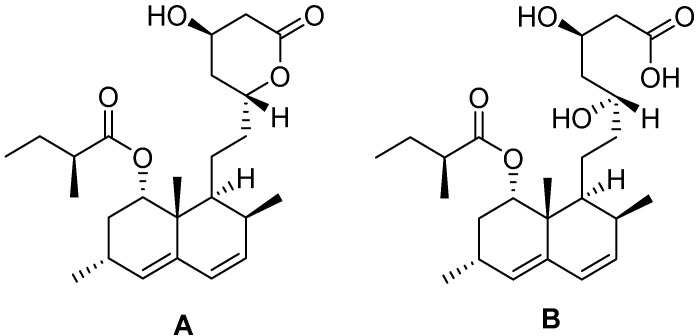
Investigated MK chemical structures: lactone LMK (**A**) and hydroxy acid AMK (**B**).

AMK acts as a competitive inhibitor of 3-hydroxy-3-methylglutaryl coenzyme A reductase (HMGCR). This enzyme catalyzes the conversion of (3S)-hydroxy-3-methylglutaryl-CoA (HMG-CoA) to mevalonic acid, the rate-limiting step in the synthesis of cholesterol. Most importantly, LMK (Figure 1), which shows the same chemical structure as the synthetic drug Lovastatin, is not active on HMGCR, but is characterized by a higher bioavailability than AMK. *In vivo*, in the liver, the hydroxyl esterase catalyzes the opening reaction of the LMK ring (Figure 1, molecule A) in which water hydrolyzes the ester bond opening, giving rise to the corresponding 5-hydroxy-1 carboxyl acid AMK (Figure 1, molecule B). AMK is the real inhibitor of HMGCR. In addition, LMK improves the quantity and quality of low-density lipoprotein (LDL) receptors on the cell membrane surface, promoting LDL degradation and facilitating cholesterol transport from blood to peripheral tissues. Interestingly, MK has demonstrated additional properties, such as it being immunomodulatory, anti-inflammatory, and antimicrobial.

Immunomodulatory activity is related to the ability to decrease the levels of intermediate metabolites in cholesterol synthesis [8]. Furthermore, MK has been proven to simultaneously promote anti-inflammatory processes via the inhibition of the synthesis of pro-inflammatory cytokines [including interleukins tumor necrosis factor-alpha (TNF-α), interferon-gamma (INF-γ)] in different cell types, by increasing the degree of phagocytosis or the production of neutrophil extracellular traps, or by inhibiting the escape mechanism of the pathogen from the phagosome [9,10].

Concerning the antimicrobial effect, MK is endowed with antifungal activity. Indeed, AMK arrests the growth of several fungal genera, including *Saccharomyces cervasie*, *Candida* spp., *Aspergillus* spp., and *Cryptococcus* spp. AMK inhibits HMGCR, depleting ergosterol synthesis, which is the cholesterol fungal counterpart. Moreover, when used in combination with other antifungal agents, such as itraconazole or fluconazole, MK is able to eliminate *Candida albicans* strains which are resistant to traditional active ingredients [3,11,12,13]. In addition, LMK demonstrated *Porphyromonas gingivalis* (at 2 µg mL^−1^) and *Fusobacterium nucleatum* (at 20 µg mL^−1^) bacteriostatic action. These are bacteria associated with the dysbiosis of oral microbiota and periodontitis [8]. 

Synthetic Lovastatin, which possesses the same chemical structure of LMK, is used in pharmaceutical formulations for cardiovascular diseases, but cannot be used in nutraceuticals and cosmetics [14]. 

Thus, given the interest of natural ingredients in nutraceutical and cosmetic products [15], this work aimed to repurpose MK as an active agent in nutraceutical/cosmetic formulations by providing a green single-step extraction approach, taking advantage of MK’s health-protective properties. Specifically, the final aim would be to exploit the MK extract as a formulation for mouth hygiene to prevent or alleviate periodontal diseases or to counter oral candidiasis in patients receiving cancer treatment [8]. 

As for the extraction, it is known that organic solvents are the primary cause of the environmental impact, posing safety risks due to their volatility and flammability. Hence, in this optimization study of MK extraction from RYR, we followed one of the twelve principles of green chemistry outlined by J.C. Warner et al., focusing on the investigation of solvents that increase extraction efficiency, minimize energy usage, and have minimal toxicity and environmental impact throughout their life cycle [16]. Firstly, we performed a comprehensive study concerning green UAE of MK from RYR. The ethanol extractive power was compared with acetonitrile (ACN), an organic solvent in which MK is very soluble. Then, the study proceeded with greener solutions by evaluating the influence of ethanol percentage in water-based mixtures, modulating temperature and extraction time in a single-factor test, and using the extraction yield as the parameter to be increased. Finally, the MK direct extraction from RYR was optimized using a representative aqueous formulation (mouthwash) under a Design of Experiment (DOE) study. This approach was explored as a fast, efficient, and more biocompatible extraction method to directly enrich the formulation with the natural molecule [17]. To evaluate the yields of extraction, a new reversed phase ultra-high-performance liquid chromatography method coupled to diode array detection (UHPLC-DAD) was developed and optimized for the determination of MK in both the LMK and AMK forms [18,19,20]. This fast UHPLC method was validated regarding accuracy, sensitivity, selectivity, linearity, and reproducibility. Finally, once the extraction conditions were optimized, the MK stability in the final mouthwash formulation was assessed.

## 2. Materials and Methods

### 2.1. Materials

ACN ≥ 99.8%, methanol ≥ 99.8%, water HPLC grade, absolute ethanol ≥ 99.8%, acetic acid 99%, lactone MK phyproof^®^ reference substance, Lovastatin sodium salt ≥ 95%, saccharin ≥ 99%, sodium benzoate BioXtra, ≥99.5%, chlorhexidine digluconate 20% aqueous solution were supplied by Merck (Darmstadt, DE, Germany). Commercial mouthwash (ingredients: aqua, xylitol, peg-40 hydrogenated castor oil, glycerin, propylene glycol, malva sylvestris, chlorhexidine digluconate, sodium benzoate, sodium saccharin, aroma, citric acid, C.I. 19,140, C.I. 42,090) was kindly provided by Ghimas S.p.A. (Bologna, Italy).

RYR bulk product as a red fine powder (particle size: NLT 95% through 80 mesh, loss on drying 5%) containing MK at 3% (*w*/*w*) of the dried weight of the powder was provided by Ghimas S.p.A. (Bologna, Italy). 

### 2.2. Standard Solutions 

For the preparation of the LMK stock solution, approximately 1.5 mg of powder was accurately weighed and solubilized in 1 mL of mobile phase composed of ACN and acid water (0.1% acetic acid) at a ratio of 50:50 (*v*/*v*). Fresh LMK stock solutions were prepared every time and immediately used for the analysis. 

The purchased AMK aqueous stock solution, 4.4 mg mL^−1^, was divided into 5 aliquots of 200 μL upon arrival and stored at −80 °C. The AMK stock solution aliquots were diluted 1:10 (0.44 mg mL^−1^) in mobile phase before use.

For the preparation of sodium benzoate and saccharin stock solutions, 1 mg of powder was solubilized in 1 mL of mobile phase to obtain 1 mg mL^−1^ concentration for both solutions. A 1:100 dilution of both the sodium benzoate and saccharin stock solution was performed before analysis by UHPLC-DAD.

A commercial chlorhexidine digluconate 20% aqueous solution was diluted (1:10^5^) with a mixture composed of ACN and formic acid 1% 20:80 (*v*/*v*) and was used as the reference standard for the identification and quantification of the active ingredient contained in the mouthwash during the stability study. 

### 2.3. UHPLC-DAD Method for the Determination of LMK and AMK Extracted from RYR 

The analyses were carried out using a Jasco X-LC (Jasco Europe, Cremella, Italy) consisting of a binary pump (3185PU), an autosampler (3059AS), a temperature controller column compartment (3067C0), a degasser module (3080DG), and a detector X-LC™ 3110 MD diode array. A reversed phase column C4 (Kromasil, Nouryon, Amsterdam, The Netherlands, 100, 50 × 3 mm, 2.5 µm) was equilibrated with a mobile phase consisting of ACN and acidic water (0.1% acetic acid) 50:50 (*v*/*v*). The column temperature was kept at 25 °C. The UV detection wavelength was set at 237 nm. The optimized chromatographic separation of LMK and AMK was performed under isocratic elution at a flow rate of 0.2 mL min^−1^ with a total runtime of 15 min. The resolution between sequential peak pairs was calculated as follows:

Rs = 2 (Rt2 − Rt1)/(W1 + W2) Rt = retention time W = chromatographic peak width

Besides the retention time, analyte identification was also assessed by comparing UV spectra acquired on standards peaks with those obtained from sample analysis.

### 2.4. Validation of the UHPLC-DAD Method for the Determination of LMK and AMK 

The proposed method for the chromatographic identification and quantification via the UHPLC-DAD of LMK and AMK was validated in terms of linearity, sensitivity, repeatability, accuracy, and recovery.

After each sample analysis, the solvent was injected twice to demonstrate the absence of any carry-over effect.

#### 2.4.1. Linearity

Standard calibration curves were prepared via the linear least-squares regression of the peak area versus the concentrations of five mixes of LMK and AMK standard solutions diluted in mobile phase in a range of 1.25–10.00 µg mL^−1^ and 0.50–8.00 µg mL^−1^, respectively. 

#### 2.4.2. Sensitivity

The limit of detection (LoD = 3 × SE/m) and the limit of quantification (LoQ = 10 × SE/m) were obtained through a statistical evaluation considering the signal deviation of the LMK and AMK standards. In particular, the LoD value was calculated by multiplying the standard error (SE) obtained from the calibration line of the standard solutions by a factor of three, and then dividing by the slope of the line. The standard error was obtained from the regression analysis of the calibration line. The LoQ value was calculated by multiplying the SE from the same calibration line by a factor of 10 and dividing by the slope of the line.

#### 2.4.3. Repeatability

Intra-day and inter-day repeatability were evaluated by analyzing standard solutions of LMK and AMK at three concentrations as follows: 1.25 µg mL^−1^, 5.00 µg mL^−1^, and 10.00 µg mL^−1^ for LMK and 0.50 µg mL^−1^, 2.00 µg mL^−1^, and 8.00 µg mL^−1^ for AMK. The UHPLC-DAD analysis was carried out in triplicate on the same day (*n* = 3) and on three different days (*n* = 3) for intra-day and inter-day repeatability, respectively. The coefficient of variation (CV) of the measurements was calculated as follow:

CV% = (Standard Deviation/Average Value) × 100

#### 2.4.4. Accuracy

The accuracy was determined by calculating the percentage of the deviation between the experimental concentrations of LMK and AMK and the nominal ones (relative error%). This was evaluated by analyzing standard solutions of LMK and AMK at three concentration levels (low, medium, and high) in the range studies: 1.25 µg mL^−1^, 5.00 µg mL^−1^, and 10.00 µg mL^−1^ for LMK and 0.50 µg mL^−1^, 2.00 µg mL^−1^, and 8.00 µg mL^−1^ for AMK. The UHPLC-DAD analysis was carried out in triplicate on the same day (*n* = 3) and in the same week on three different days (*n* = 3) for intra-day and inter-day accuracy, respectively. Relative error% between the measured value and nominal concentration was calculated using the following formula:

Relative Error% = absolute error/measured value × 100 = [(measured Value − nominal Value)/measured value] × 100

#### 2.4.5. Recovery

The recovery represents the percentage of analyte recovered by applying the developed method and allows for the determination of analyte losses, therefore expressing the accuracy of both the extraction and UHPLC-DAD determination method. Furthermore, recovery was calculated by comparing the amount of total MK extracted with the HPLC-DAD method and the amount declared by the company that provided the RYR powder. The recovery% was calculated as the mean value of three different extractions ± standard deviation. 

### 2.5. UAE of MK from RYR Using Different Solvents: Comparison between ACN, Ethanol, and Water

The UAEs were carried out using Elmasonic S60H sonicator, (Elma, Singen, DE, Germany) (ultrasound power of 250 W), and the samples were centrifuged using a CL10 centrifuge (Thermo Scientific, Waltham, MA, USA).

#### 2.5.1. Extractions by ACN and Ethanol

About 5 mg of RYR powder was weighted in a falcon tube. A volume equal to 5 mL of ACN and ethanol was applied for the MK extraction, respectively. The suspension was vortexed for 1 min and subjected to UAE in the ultrasound bath at 25 °C for 30 min. Then, the sample was centrifuged (5 min, 4000 rpm) and the supernatants were volumetrically collected. The UAE cycle was repeated three times. All the extracts were collected, and 4 mL of the total volume was withdrawn and dried with a rotatory evaporator. The obtained powder was solubilized in 4 mL of mobile phase ACN/H_2_O (50:50) (*v*/*v*) (0.1% acetic acid). The solubilized extracts were filtered by a nylon syringe filter (0.22 µm) before being analyzed by the UHPLC-DAD system. 

#### 2.5.2. Extractions by Ethanol and Water Mixtures 

The study was carried out by testing solutions composed of water and ethanol containing different percentages of organic solvent (0%, 50%, and 99%). The efficacy of the three extracting solutions was evaluated at different temperatures (25 °C, 50 °C, and 80 °C) and times (10, 30, and 60 min). In order to carry out the extraction under the reported conditions, 10 different samples were prepared by diluting about 1 mg of RYR powder in 1 mL of solvent. Each suspension was vortexed for 1 min. The samples were then subjected to UAE under the different experimental conditions reported in the worksheet (Table S1). Then, after each UAE cycle, the extracts were centrifuged at 4000 rpm at 25 °C for 5 min, and the supernatants were collected after each extraction (*n* = 3) in a falcon tube and stored in the fridge (4 °C). The total extract was transferred in a round bottom flask and dried by a rotatory evaporator. The dried extract obtained for each sample was then solubilized in 1 mL of mobile phase and filtered by a nylon syringe filter (0.22 µm) before the analysis in the UHPLC-DAD system. LMK and AMK concentrations were determined using the calibration curves equations, and, considering the dilution factor, their content in the extract was determined.

### 2.6. DOE for the Optimization of LMK and AMK Extraction from RYR Using a Standard Mouthwash Formulation 

To investigate the best UAE conditions of MK from RYR by using a commercial mouthwash as the extraction phase (composition reported in Section 2) a DOE study was conducted. MODDE^®^ Pro 13 Software v.UT-MS-0138 (Sartorius Data Analytics, Goettingen, DE, Germany) was exploited for this study. For the DOE, an optimization design, D-optimal (3 levels), was selected. The total extraction yield of MK, determined by UHPLC-DAD analysis, was selected as the response to be maximized. Three main multilevel factors were considered as follows: temperature, time, and quantity of sample powder. Each factor was evaluated at three levels as follows: temperature = 25, 45, 80 °C; time = 10, 30, 45 min; quantity of RYR sample powder = 5, 50, 100 mg in 1 mL of mouthwash (matrix/solvent ratio: 5 mg mL^−1^, 50 mg mL^−1^, 100 mg mL^−1^).

Furthermore, the worksheet describing the experiments to be performed was generated producing 15 experiments and 3 center points (Table S2). Then, extractions were carried out by weighting the RYR powder and by adding 1 mL of mouthwash. Each suspension was then vortexed for 1 min. After stirring, the sample was ultra-sonicated under the time and temperature conditions suggested by the DOE. After each UAE cycle, the samples were centrifuged for 5 min at 4000 rpm, then the supernatant was withdrawn and collected in a round bottom flask. This extraction procedure was repeated 3 times.

The extracts were then dried by a rotatory evaporator and solubilized with 2 mL of mouthwash to verify by the UHPLC-DAD analysis also the interference between the MK forms and the mouthwash components.. Each of the obtained solutions was filtered with a nylon syringe filter (0.22 µm) and then diluted 1:10 using the mobile phase. Each extract was injected twice in the UHPLC-DAD system.

The chromatographic peak areas determined for LMK and AMK were interpolated into the standard calibration curves to evaluate the extracted quantities. Therefore, the yields values obtained for the 18 extractions were compared by applying the following equations:

LMK% = mass LMK (µg) × 100/RYR powder (µg)(1)

AMK% = mass AMK (µg) × 100/RYR powder (µg)(2)

Total MK% = % of LMK + % of AMK found in RYR powder(3)

MK yield = (% of Total MK found × 100)/% of MK declared in RYR powder(4)

Then, the DOE worksheet was filled with all the data obtained for the MK yields (Equation (4)). Results were processed again by MODDE^®^ Pro 13 software, and the optimal extraction parameters for sample preparation were determined. 

Finally, to study the stability of MK in the mouthwash extract, three independent extractions from RYR samples were performed following the DOE-optimized conditions. Each extract was stored at room temperature for 10 days (t10) and then analyzed twice in the UHPLC-DAD system under the chromatographic conditions described in Section 2.3 (t0). The degradation rate of LMK into AMK was estimated by the following equation:

% LMK hydrolysis = 100 − [(Peak area LMK _t10_ − Peak area LMK _t0_) × 100]

## 3. Results and Discussion

Recently, the antimicrobial, anti-inflammatory, and immunomodulatory actions of statins have been considered in the periodontal field. In particular, various studies reported that statins can improve the clinical profile of periodontitis, including gingival bleeding and loss of bone density in the teeth [10,21]. 

In line with these findings, MK contained in RYR, whose lactone form shares the same chemical structure of the drug Lovastatin, has been proven to exhibit antimicrobial and anti-inflammatory properties, in addition to the previously known anti-hypercholesterolemic activity. Thus, in this work, the feasibility of MK green extraction from RYR was explored by the development of a new UHPLC-DAD method for AMK and LMK determination in the different extracts: organic solvents, aqueous solutions, and commercial mouthwash. 

Moreover, the enriched mouthwash with MK might be applied as an antiseptic formulation for mouth hygiene, specifically in cases of periodontal disease or oral candidiasis.

Specifically, the UAE method was selected for its ability to extract active substances via the cavitation effect, thus enhancing contact between the active substances and the solvent for a more effective extraction [21,22,23]. In addition, when considering a large-scale industrial extraction, the ultrasound apparatus is less expensive and easier to be used in comparison to the microwave apparatus [24].

In order to determine the LMK and AMK extraction yield in the different conditions, a UHPLC method with DAD detection was optimized and validated.

Firstly, it was proven that ethanol has an extraction efficacy comparable to traditional organic solvents such as ACN. This evidence justified the extractive study using a mixture of ethanol and water, evaluating the influence of temperature, time, and solvent percentage on the extraction yield of MK from RYR in a single-factor experiment. Then, since the results for water extraction in terms of MK yield were encouraging (yield% = 85.9 ± 1.2), it was directly used a mouthwash formulation as the extraction solvent. In this way, it was possible to have both a biocompatible and environmentally friendly extraction and a simplified, less laborious single-step procedure, by which extraction and dissolution in the mouthwash occur simultaneously in an accurate and reproducible manner.

Since traditional univariate optimization is unable to assess individual and combined interactions between process parameters, statistical modeling techniques such as response surface methodology (RSM) are better and increasingly more common approaches for process optimization [21,25,26]. Therefore, mouthwash extraction parameters (time, temperature, and solvent-to-solid ratio) were optimized by a DOE study. 

### 3.1. UHPLC-DAD Chromatographic Method Optimization for the Determination of LMK and AMK

Several analytical methods based on HPLC with UV detection for the quantification of monacolins, in both the acid and lactone forms, in RYR have already been described in the literature [19,27,28,29,30]. However, aiming to improve resolution and time of analysis, a new fast UHPLC-DAD method was developed. Separative conditions were optimized to avoid any interferences by the other RYR components and the ingredients of the water-based formulations, selectively determining the lactone and acid forms of MK in a very short and convenient time.

In more detail, preliminary chromatographic analyses were performed by a HPLC-DAD Agilent 1260 Infinity LC System (Santa Clara, CA, USA) with a C4 column (Kromasil, Nouryon, Amsterdam, The Netherlands 100, 50 × 3 mm, 2.5 µm). Then, the chromatographic conditions were optimized based on selectivity, peak resolution, and elution time in a RP-UHPLC-DAD system (JascoX-LC, Jasco Europe, Cremella, Italy). The effects on the chromatographic analysis, determined by the type of organic modifier (methanol, ACN), its percentage in the mobile phase composition, the grade of acidity, and the type of stationary phase (C18-C8-C4), were evaluated (data not reported). ACN was chosen as the organic modifier due to less viscosity and the minor back pressure of the mobile phase when compared to methanol. Firstly, the analyses were performed using a mobile phase composed of ACN and water 80:20 (*v*/*v*) at a flow rate of 0.4 mL min^−1^, with a C18 column (Kinetex, Phenomenex, Torrance, CA, USA, 50 × 2.1, 1.7 µm) as the stationary phase, which is widely reported in the literature as suitable for the chromatographic separation of monacolin species [19,29]. 

However, in these conditions, acid and lactone MK eluted too early and the acid form peak was not completely resolved from the front. Decreasing the ACN percentage from 80 to 50, the MK chromatographic peaks were well separated and the acid and lactone forms eluted at retention times of 0.88 min and 1.59 min, respectively. Then, the UHPLC analyses were also focused on the separation of the two MK forms from the mouthwash excipients. In these chromatographic conditions, saccharin and sodium benzoate eluted too early, even decreasing the ACN percentage from 50 to 20, meaning that a satisfying resolution of the excipients was not obtained. Therefore, to improve the separation, a C8 column (Kinetex, Phenomenex, Torrance, CA, USA, 50 × 2.1 mm, 2.6 µm) was tested, but the resolution of the peaks was not improved even when the ACN amount was further decreased to 30% in the mobile phase. C18 and C8 columns demonstrated to be too lipophilic and less efficient in the separation of the mouthwash excipients from the analytes. Then, a C4 column (Kromasil, Nouryon, Amsterdam, The Netherlands 100, 50 × 3 mm, 2.5 µm) was selected. A good resolution was attained by using a mobile phase consisting of ACN and water (0.1% of acetic acid) 50:50 (*v*/*v*) at a flow rate of 0.2 mL min^−1^, with UV detection at 237 nm. Under these chromatographic conditions, the retention times of saccharin and sodium benzoate were 1.2 min and 2.3 min, respectively, while AMK and LMK eluted at 6.7 min and at 11.7 min, respectively. Moreover, the reduction in the flow rate to 0.2 mL min^−1^ ensured less solvent consumption in a chromatographic run (15 min) that is faster than the previously reported HPLC methods which require at least 20 min [19,29]. 

In addition, a UHPLC-DAD method (conditions reported in par. 4 of SI) was developed to determine chlorhexidine digluconate in the MK mouthwash extract for verifying its stability during DOE-optimized conditions. 

### 3.2. Validation of the UHPLC-DAD Method for the Determination of LMK and AMK

#### 3.2.1. Linearity and Sensitivity

The standard calibration curves for LMK and AMK were obtained by plotting the peak area of five incremental standard mix solutions versus the corresponding concentrations in the ranges 1.25–10.00 µg mL^−1^ and 0.50–8.00 µg mL^−1^, respectively. Satisfactory correlation coefficients (R^2^ > 0.999) were obtained for both analytes’ calibration curves (Table 1, par. 3.1. SI, Figure S2). 

The LMK LoD and LoQ values were found to be 0.211 ± 0.089 µg mL^−1^ and 0.703 ± 0.297 µg mL^−1^, respectively. AMK LoD (0.169 ± 0.003 µg mL^−1^) and LoQ (0.533 ± 0.011 µg mL^−1^) values were obtained. 

**Figure 2:**
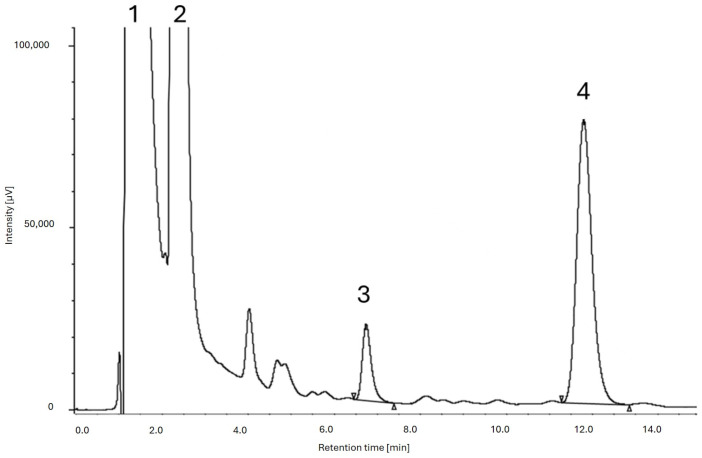
UHPLC-DAD analysis of the RYR water formulation extract. Chromatographic conditions: C4 stationary phase, mobile phase: ACN and water (0.1% of acetic acid) 50:50 (*v*/*v*), flow rate 0.2 mL min^−1^; detection at 237 nm: (1) saccharin, (2) sodium benzoate, (3) AMK, and (4) LMK.

LoD and LoQ values demonstrated that the UHPLC-DAD method was more sensitive when compared to the literature data [29]. Indeed, this method was also found to be twice more sensitive in terms of LoQ values in comparison to other methods previously reported [30]. Moreover, the developed UHPLC-DAD method showed a satisfactory resolution between two sequential peak pairs (Rs = 3.76) also in the presence of the mouthwash ingredients (Figure 2). 

#### 3.2.2. Repeatability and Accuracy

The determination of intra-day and inter-day repeatability was carried out on standard solutions. The variation coefficients (CV%) for the intra-and inter-day precision of AMK and LMK standard solutions, tested at three different concentration levels, were found to be acceptable since the CV% average value were less than 15% (Table 2), thus in agreement with Food and Drug Administration guidelines [31]. 

Accuracy (less than 15%), at the three different concentration levels, was calculated as the relative error between the experimental concentrations of LMK and AMK and the nominal concentrations (Table 2), thus confirming the closeness between experimental and true values [31].

#### 3.2.3. Recovery

The recovery, expressed as yield% (amount of MK extracted/amount of MK declared%), resulted to be 97.6 ± 2.0% for the ethanol/water mixture and 86.6 ± 0.4% for the mouthwash formulation. The high level of recovery confirmed that the water-based mouthwash can be considered a convenient green solvent for the extraction of MK.

### 3.3. MK UAE

The main goal of this work consisted of the optimization of the UAE of MK from RYR using non-toxic and biocompatible solvents following one of the twelve principles of green chemistry [16]. The extraction of MK (AML and LMK forms) from RYR bulk powder using organic solvent (particularly with ACN, methanol, and ethanol) was already reported in previous studies (both with classical extraction systems and UAEs) [19,30,32,33].

Therefore, the ethanol extractive efficiency was firstly compared to ACN following the procedure described in par. 2.5.1. Data obtained regarding the MK extraction yields performed with the two solvents showed a recovery of 80.7 ± 0.1% with ethanol. Furthermore, the latter showed to be only slightly less efficient than ACN (85.5 ± 0.2%), reporting a 5% difference in recovery in the same extractive conditions (time, temperature, and matrix/solvent ratio). Since ACN and ethanol demonstrated to be comparable in terms of MK yield, ethanol was selected as a suitable and green solvent for evaluating the influence of temperature, time, and solvent percentage in the extraction yield of MK from RYR. 

In the literature, MK extraction from RYR has been investigated using variable ethanol concentrations (i.e., 40%, 50%, 60%, 70%, 80%, and 95% (*v*/*v*)) at two temperature conditions (i.e., room temperature and 60 °C); in the same study, the extractions were conducted by weighting RYR powder in an Erlenmeyer of 50 mL containing the ethanol solution, then the suspension was agitated in a shaker incubator for 2 h [32].

Therefore, in our current knowledge, there is only one study in which MK extraction was optimized by UAE using a 15 mL ethanol/water mixture (*v*/*v*) (75:25) for 150 mg of RYR powder and in which the procedure demonstrated the same extraction efficacy (recovery around 98%) of a mixture as ACN–buffer (pH = 7) [19].

Hence, aiming to enlarge the study about the ethanol and water mixture, 10 UA MK extractions from RYR at modulating times and temperatures in different mixture percentages of ethanol and water in single-factor experiments were carried out. For each extractive condition, results were analyzed in terms of the MK total yield (%) and as the ratio between the discovered amounts of lactone and acid MK forms (%). Data also demonstrated that time and temperature did not affect the total MK yield when the extractive solvent mix contained a percentage of ethanol higher than 50%. On the contrary, when increasing the percentage of water in the extractive mixture (more than 50%), temperature became more influent to improve the MK yield. Overall, as it is reported in Table S1 and Figure S1, the higher MK yield rates were found to be 97.6 ± 2.0% and 96.6 ± 0.8% by using, respectively, 50:50 (*v*/*v*) water and ethanol at 50 °C for 30 min and 1:99 (*v*/*v*) water and ethanol at 80 °C for 60 min, with the solid/solvent ratio equal to 1 mg mL^−1^.

In view of researching a greener and more biocompatible extractive strategy, the data obtained in the extractions with 100% water were evaluated, and it was found that, at room temperature (25 °C), even when increasing the time of extraction (60 min), there was no improvement in the MK yield (around 50%). Interestingly, when increasing the temperature up to 80 °C, a comparable and acceptable MK yield (higher than 85%) was obtained after only 10 min. 

Even if in water-based extractions, the hydrolysis rate is increased and MK is mainly present as AMK; the presence of AMK is advantageous for topical action. 

These data were found to be encouraging for investigating the feasibility of single-step MK extraction from RYR using a commercial water-based mouthwash.

### 3.4. DOE for the Optimization of LMK and AMK Extraction from RYR Using a Mouthwash 

**Figure 3:**
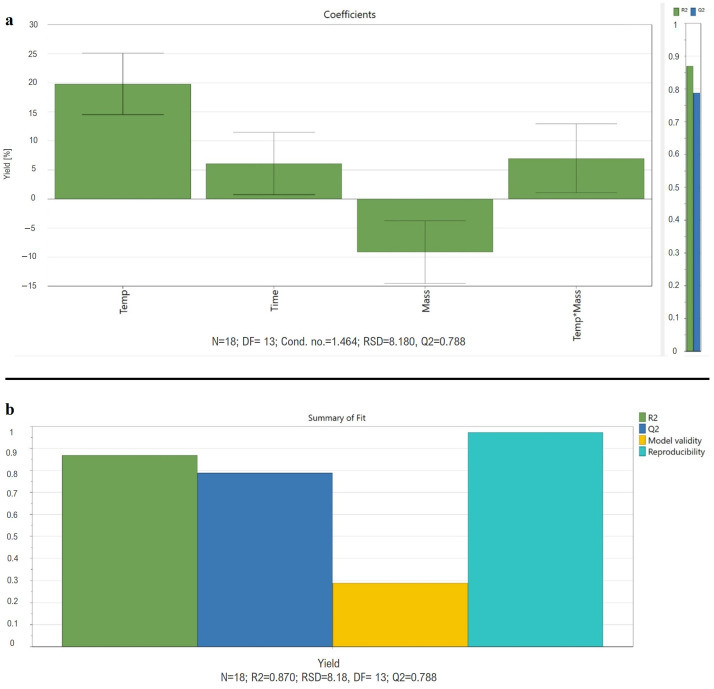
(**a**) Coefficient plot reports the primary effects of temperature, time, mass, and the interaction effect of temperature × mass for the MK yield of extraction; (**b**) Summary of Fit reports values regarding R^2^, Q^2^, model validity, and reproducibility of the performed DOE.

The optimization of the UAE of MK from RYR using a water-based mouthwash was performed through a DOE study. Exploiting MODDE^®^ Pro 13 Software (Sartorius Data Analytics), a three-level D-optimal design in the optimization DOE study was set and the best extraction conditions were evaluated. The total yield of MK was selected as the response to be maximized since the two forms are both active. The sonication temperature, time, and the ratio quantity of the RYR powder/solvent volume were selected as factors to be evaluated. The performed center points demonstrated that, in the experimental domain, the experimental error was acceptable as it was less than 30% of the response variation. The response distribution showed a bell-shaped normal distribution; therefore, it did not need to be transformed according to the skewness test. The analysis of the coefficient plot evaluated the primary effects of the quantity of RYR powder, the sonication times and temperatures, the interaction effects (mass × time, temperature × mass, time × temperature), and the quadratic effects (time × time, temperature × temperature and mass × mass of each factor). To improve the regression (R^2^) and prediction (Q^2^) parameters of the statistical model, the interaction effects of temperature × time and mass × time and the quadratic effects of time × time and mass × mass were eliminated as they did not appear to be significant (Figure 3a).

**Figure 4:**
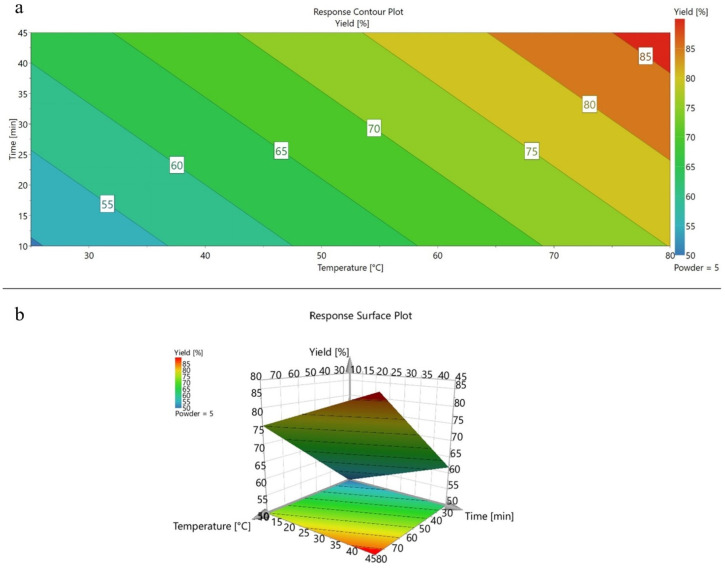
Three-dimensional response contours of MK extractions with time, temperature, and yield at a solid/solvent 5 mg mL^−1^ ratio.

The coefficient regression model (R^2^ = 0.870) demonstrates the strength of the linear relationship, while the prediction coefficient of the model (Figure 3b) confirmed the goodness of the model to be applies for future predictions (Q^2^ = 0.788) (Model Validity = 0.290). The 2D response contour of the total yield of MK was obtained by plotting time (x axis) and temperature (y axis). The color scale reported in 2D and 3D response contour (Figure 4a,b), highlighted the best extraction conditions in the red area. It was clear that the parameters ensuring the higher extraction yield of total MK in the studied experimental domain were temperature = 80 °C, time of extraction = 45 min, and mass = 5 mg of RYR powder in 1 mL of mouthwash (5 mg mL^−1^) 

In comparison to previous works, this optimization study allowed us to reduce the time for each UAE cycle (45 min) and also to use a smaller amount of RYR powder (5 mg) and solvent (1 mL) [19,30,32]. Moreover, in a single procedure, the MK extraction and the enrichment of the final formulation were combined.

**Figure 5:**
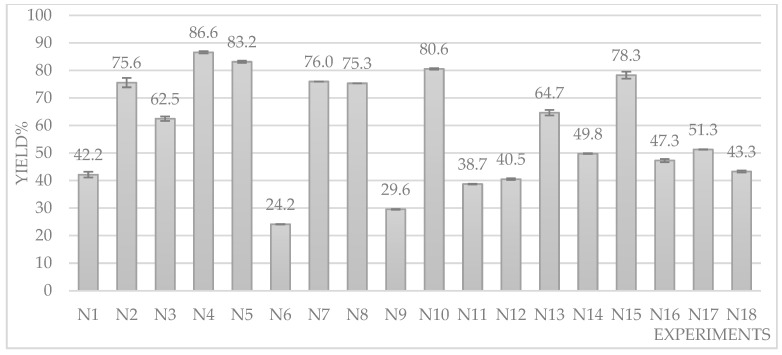
LMK + AMK yields after UAE under DOE experimental conditions.

By using the data collected during the DOE study, the total MK yield was calculated and reported in Figure 5, showing an important variation in yield, with a minimum of 24.2 ± 0.1% up to over 86.6 ± 0.4%, depending on the extraction conditions. 

The optimal conditions in terms of the total MK yield UAE were found to be in N4: 80 °C for 45 min with 5 mg mL^−1^ of RYR powder/solvent (Figure 5, Table S2). This result is in line with the data reported in the literature [27]. 

Hence, since the yield% obtained using the mouthwash as extraction solvent (86.6 ± 0.4%) was shown to be comparable to that obtained using 50:50 ethanol/water (97.6 ± 2.0%), a quick process to prepare an oral formulation based on MK directly extracted from the RYR was optimized using a commercial water-based mouthwash.

### 3.5. Stability of Mouthwash Final Formulation

In order to exclude the hydrolysis of chlorhexidine digluconate, a common ingredient present in the mouthwash formulation, during the extraction procedure, its stability was verified in the adopted best extraction conditions (80 °C for 45 min with 5 mg mL^−1^ of RYR powder/solvent). Aqueous solutions of chlorhexidine are mostly stable within the pH range of 5–8. The hydrolysis that leads to the formation of the toxic p-chloroaniline is reduced at room temperature, but it is increased above 100 °C, especially at alkaline pHs [34].

In light of this, the attention was focused on the potential degradation of chlorhexidine digluconate. It was verified that increasing the temperature did not induce its conversion into the toxic p-chloroaniline product during the optimized extraction of MK from RYR by mouthwash. Thus, chlorhexidine digluconate solution was subjected to the three cycles in the ultrasound bath at 80 °C for 45 min and analyzed under the UHPLC conditions, as described in par. 4 of SI.

The UHPLC analysis did not reveal an additional peak corresponding to the degradation product, and the recovery of chlorhexidine digluconate, calculated considering the peak area before and after the thermal process, seemed to be higher than 99%. Hence, chlorhexidine digluconate was considered stable in the final formulation. 

Then, the stability of MK in the mouthwash extract was studied by analyzing the results obtained from three independent extractions of RYR samples carried out using the DOE-optimized conditions. The samples stored at room temperature for 10 days (t = 10) were then analyzed under the optimized UHPLC-DAD conditions (par. 2.3). The total amount of MK was unchanged. Only about 7% of LMK was hydrolyzed to the acid form, which, however, represents the active form. 

## 4. Conclusions

The present study aimed to evaluate green methods to extract MK as LMK and AMK from RYR to finally enrich water-based formulations by performing a single-step extraction.

For this purpose, a new UHPLC-DAD method was developed and validated to determine the acid and lactone form of MK after the UAEs. The development and validation of an easy separative UHLPC-DAD method based on a C4 RP column and isocratic elution provided the best resolution of LMK and AMK at decreased elution times. This UHPLC method showed better performance and required a smaller quantity of solvents in comparison to other HPLC methods reported in the literature.

Moreover, the study about green UAEs was enlarged using a ethanol and water mixture and by evaluating a wider range of experimental conditions, such as % ethanol (99–0%) and temperature (25–80 °C), and, in the meantime, the powder/solvent ratio was optimized, reducing the required RYR matrix/solvent ratio (5 mg mL^−1^).

Hence, starting from a “one-factor-at-a-time” approach, the influence of temperature, time, and solvent percentage in the extraction yield of MK from RYR was studied using greener ethanol and water mixtures. Regarding UAEs with 100% water, the data demonstrated that by increasing the temperature up to 80 °C for 10 min, a comparable and acceptable MK yield (higher than 85%) demonstrating LMK hydrolysis to AMK (LMK/AMK = 2.06 ± 8.57 × 10^−5^) was obtained. In this case, hydrolysis is favorable since AMK is the preferred form for topical activity. Finally, the work focused on a faster method to combine MK extraction and the enrichment of a water-based formulation in a single step. Via a DOE study, the optimal conditions for the UAE of MK from RYR by a mouthwash formulation were found to be 80 °C for 45 min at a 5 mg mL^−1^ solid/liquid ratio, resulting in an 86.6 ± 0.4% MK yield. Thus, a final stable formulation containing MK (130.00 ± 0.60 µg mL^−1^) was obtained with the advantage of excluding organic solvents and reducing production processes in a green approach, proving that LMK and AMK at active concentrations can be added in water-based formulations by a single-step extraction. Hence, further investigations could focus on the application of the prepared water-based formulation in the periodontal field and to counteract oral candidiasis. 

## Figures and Tables

**Table 1:** Linearity parameters for AMK and LMK calibration curves by UHPLC-DAD analyses.

Analyte	Calibration Curve	R^2^
LMK	y = (169,743 ± 7667.00)x + (14,678 ± 3846.66)	0.999
AMK	y = (146,940 ± 243.00)x + (762 ± 1699.13)	0.999

**Table 2:** Table reports the intra-day and inter-day variation coefficients and accuracy of LMK and AMK. CV = variation coefficient; RE = relative error.

LMK Concentration µg mL^−1^	Intra-Day Repeatability CV%	Inter-Day Repeatability CV%	Intra-Day Accuracy RE%	Inter-Day Accuracy RE%
1.25	0.294	0.10	4.30	−2.41
5.00	0.348	1.32	−0.92	−2.54
10.00	0.788	2.99	0.20	−1.98
AMK concentration µg mL^−1^	Intra-day repeatability CV%	Inter-day repeatability CV%	Intra-day accuracy RE%	Inter-day accuracy RE%
0.50	0.384	0.839	6.71	7.26
2.00	0.138	1.800	−0.79	−2.49
8.00	0.104	0.042	0.43	0.49

## Data Availability

The original contributions presented in the study are included in the article/supplementary material, further inquiries can be directed to the corresponding author.

## Supplementary Materials

The following supporting information can be downloaded at https://www.mdpi.com/article/10.3390/foods13162509/s1, Table S1: table reports ethanol and water mixture extraction experiments. The reported water percentages are intended in ethanol mixtures; Figure S1: the graph reports the extractive condition results analyzed in terms of total yield of MK (dark gray) (%) and as ratio between the lactone and acid MK forms found amounts (gray) (%); Table S2: table reports the worksheet obtained with MODDE^®^ Pro 13 describing the experiments to be performed and the related results. Figure S2: reports the LMK and AMK calibration curves.

## References

[1] Zhang Y., Chen Z., Wen Q., Xiong Z., Cao X., Zheng Z., Zhang Y., Huang Z. (2020). An Overview on the Biosynthesis and Metabolic Regulation of Monacolin K/Lovastatin. Food Funct..

[2] Cicero A.F.G., Fogacci F., Banach M. (2019). Red Yeast Rice for Hypercholesterolemia. Methodist Debakey Cardiovasc. J..

[3] Xiong Z., Cao X., Wen Q., Chen Z., Cheng Z., Huang X., Zhang Y., Long C., Zhang Y., Huang Z. (2019). An Overview of the Bioactivity of Monacolin K / Lovastatin. Food Chem. Toxicol..

[4] Red Yeast Rice Market Insight, Evaluating Share, Report 2023–2032. https://www.polarismarketresearch.com/industry-analysis/red-yeast-rice-market.

[5] Wen Q., Cao X., Chen Z., Xiong Z., Liu J., Cheng Z., Zheng Z., Long C., Zheng B., Huang Z. (2020). An Overview of Monascus Fermentation Processes for Monacolin K Production. Open Chem..

[6] Global Market Study on Red Yeast Rice: Sales to Balloon 2.4X Over Next Ten Years. https://www.persistencemarketresearch.com/market-research/red-yeast-rice-market.asp.

[7] Suraiya S., Kim J.-H., Tak J.Y., Siddique M.P., Young C.J., Kim J.K., Kong I.-S. (2018). Influences of Fermentation Parameters on Lovastatin Production by *Monascus purpureus* Using *Saccharina japonica* as Solid Fermented Substrate. LWT.

[8] Kamińska M., Aliko A., Hellvard A., Bielecka E., Binder V., Marczyk A., Potempa J., Delaleu N., Kantyka T., Mydel P. (2019). Effects of Statins on Multispecies Oral Biofilm Identify Simvastatin as a Drug Candidate Targeting Porphyromonas Gingivalis. J. Periodontol..

[9] Graziano T.S., Cuzzullin M.C., Franco G.C., Schwartz-Filho H.O., de Andrade E.D., Groppo F.C., Cogo-Müller K. (2015). Statins and Antimicrobial Effects: Simvastatin as a Potential Drug against Staphylococcus Aureus Biofilm. PLoS ONE.

[10] Bergman P., Linde C., Pütsep K., Pohanka A., Normark S., Henriques-Normark B., Andersson J., Björkhem-Bergman L. (2011). Studies on the Antibacterial Effects of Statins—In Vitro and In Vivo. PLoS ONE.

[11] Zhou Y., Yang H., Zhou X., Luo H., Tang F., Yang J., Alterovitz G., Cheng L., Ren B. (2018). Lovastatin Synergizes with Itraconazole against Planktonic Cells and Biofilms of Candida Albicans through the Regulation on Ergosterol Biosynthesis Pathway. Appl. Microbiol. Biotechnol..

[12] Macreadie I.G., Johnson G., Schlosser T., Macreadie P.I. (2006). Growth Inhibition of Candida Species and Aspergillus Fumigatus by Statins. FEMS Microbiol. Lett..

[13] Lorenz R.T., Parks L.W. (1990). Effects of Lovastatin (Mevinolin) on Sterol Levels and on Activity of Azoles in Saccharomyces Cerevisiae. Antimicrob. Agents Chemother..

[14] Hasan F., Ikram R., Simjee S.U., Iftakhar K., Asadullah K. (2019). Effectiveness of Simvastatin 1% Oral Gel and Mouthwash Used as an Adjunct Treatment of Scaling and Root Planning in the Treatment of Periodontal Diseases. Pak. J. Pharm. Sci..

[15] Rybczyńska-Tkaczyk K., Grenda A., Jakubczyk A., Kiersnowska K., Bik-Małodzińska M. (2023). Natural Compounds with Antimicrobial Properties in Cosmetics. Pathogens.

[16] Anastas P.T., Warner J.C. (1998). Green Chemistry: Theory and Practice.

[17] Xie L., Zhu G., Shang J., Chen X., Zhang C., Ji X., Zhang Q., Wei Y. (2021). An Overview on the Biological Activity and Anti-Cancer Mechanism of Lovastatin. Cell. Signal..

[18] Montanari S., Davani L., Tumiatti V., Dimilta M., Gaddi A.V., De Simone A., Andrisano V. (2021). Development of an UHPLC-Diode Arrays Detector (DAD) Method for the Analysis of Polydatin in Human Plasma. J. Pharm. Biomed. Anal..

[19] Theunis M., Naessens T., Verhoeven V., Hermans N., Apers S. (2017). Development and Validation of a Robust High-Performance Liquid Chromatographic Method for the Analysis of Monacolins in Red Yeast Rice. Food Chem..

[20] Davani L., Terenzi C., Tumiatti V., De Simone A., Andrisano V., Montanari S. (2022). Integrated Analytical Approaches for the Characterization of Spirulina and Chlorella Microalgae. J. Pharm. Biomed. Anal..

[21] Kraboun K., Tochampa W., Jittrepotch N., Rojsuntornkitti K., Chatdamrong W., Kongbangkerd T. (2017). Optimization of Ultrasonic-Assisted Extraction for Monacolin K, Antioxidant Activity, Pigment and Citrinin of Monascal Waxy Corn by Response Surface Methodology. Food Appl. Biosci. J..

[22] Carvalho J.C., Oishi B.O., Woiciechowski A.L., Pandey A., Babitha S., Socco C.R. (2007). Effect of Substrates on the Production of Monascus Biopigments by Solid-State Fermentation and Pigment Extraction Using Different Solvents. IJBT.

[23] Yang S., Zhang H., Li Y., Qian J., Wang W. (2005). The Ultrasonic Effect on Biological Characteristics of *Monascus* sp.. Enzym. Microb. Technol..

[24] Ciriminna R., Carnaroglio D., Delisi R., Arvati S., Tamburino A., Pagliaro M. (2016). Industrial Feasibility of Natural Products Extraction with Microwave Technology. ChemistrySelect.

[25] Zhou G., Fu L., Li X. (2015). Optimisation of Ultrasound-Assisted Extraction Conditions for Maximal Recovery of Active Monacolins and Removal of Toxic Citrinin from Red Yeast Rice by a Full Factorial Design Coupled with Response Surface Methodology. Food Chem..

[26] Davani L., Tassinari E., Chiaberge S., Siviero A., Serbolisca L., Tumiatti V., Terenzi C., De Simone A., Andrisano V., Montanari S. (2023). Safety Issues in Nutraceutical Exploitation of *Chlorella vulgaris*, *Arthrospira platensis* and *Scenedesmus* sp. Microalgae. J. Food Compos. Anal..

[27] Wu C.-L., Kuo Y.-H., Lee C.-L., Hsu Y.-W., Pan T.-M. (2011). Synchronous High-Performance Liquid Chromatography with a Photodiode Array Detector and Mass Spectrometry for the Determination of Citrinin, Monascin, Ankaflavin, and the Lactone and Acid Forms of Monacolin K in Red Mold Rice. J. AOAC Int..

[28] Heber D., Lembertas A., Lu Q.Y., Bowerman S., Go V.L. (2001). An Analysis of Nine Proprietary Chinese Red Yeast Rice Dietary Supplements: Implications of Variability in Chemical Profile and Contents. J. Altern. Complement. Med..

[29] Li Y.-G., Zhang F., Wang Z.-T., Hu Z.-B. (2004). Identification and Chemical Profiling of Monacolins in Red Yeast Rice Using High-Performance Liquid Chromatography with Photodiode Array Detector and Mass Spectrometry. J. Pharm. Biomed. Anal..

[30] Huang H.-N., Hua Y.-Y., Bao G.-R., Xie L.-H. (2006). The Quantification of Monacolin K in Some Red Yeast Rice from Fujian Province and the Comparison of the Other Product. Chem. Pharm. Bull..

[31] Bioanalytical Method Validation Guidance for Industry. https://www.fda.gov/regulatory-information/search-fda-guidance-documents/bioanalytical-method-validation-guidance-industry.

[32] Singgih M., Saraswaty V., Ratnaningrum D., Priatni S., Damayanti S. (2014). The Influence of Temperature and Ethanol Concentration in Monacolin K Extraction from Monascus Fermented Rice. Procedia Chem..

[33] Ajdari Z., Ebrahimpour A., Abdul Manan M., Hamid M., Mohamad R., Ariff A.B. (2011). Assessment of Monacolin in the Fermented Products Using Monascus Purpureus FTC5391. J. Biomed. Biotechnol..

[34] Chlorexidine Digluconate 20% Aqueous Solution Sigma Prod. No. C9394. https://www.sigmaaldrich.com/IT/it/product/sigma/c9394.

