# Supplementary material for: Design of Experiments and Optimization of Monacolin K Green Extraction from Red Yeast Rice by Ultra-High-Performance Liquid Chromatography
Source: Foods. 2024 Aug 11;13(16):2509. doi: 10.3390/foods13162509 (PMC11353663; doi:10.3390/foods13162509)
Supplement: Supplementary file 1 [file foods-13-02509-s001.zip › foods-3126392-supplementary.pdf]

Supplementary information

Design of experiments and Optimization of Monacolin K Extraction from Red Yeast Rice by Ultra High Performance Liquid Chromatography (UHPLC)

Lara Davani[a], Cristina Terenzi[a], Angela De Simone[b], Vincenzo Tumiatti[a], Vincenza Andrisano[a], Serena Montanari[a]*

[a]Department for Life Quality Studies, University of Bologna, Corso D'Augusto 237, 47921 Rimini, Italy;

[b]Department of Drug Science and Technology University of Torino, via P. Giuria 9, 10125 Torino, Italy;

*corresponding author

Serena Montanari

Department for Life Quality Studies

University of Bologna

Corso D'Augusto 237

47921 Rimini, Italy

e-mail: serena.montanari5@unibo.it

1. Ultrasound assisted extraction of MK from RYR using different solvents: comparison between acetonitrile, ethanol and water.

The study was carried out by testing solutions composed by water and ethanol containing different percentage of organic solvent (0%, 50% and 99%). The efficacy of the three extracting solutions was evaluated at different conditions of temperatures (25 °C, 50 °C and 80°C) and time (10, 35 and 60 min). Briefly, for 10 samples about 1 mg of RYR powder was quantitatively transferred in a Eppendorf tube and then 1 mL of solvent was added. Each suspension was vortexed for 1 min. The samples were then subjected to UAE under the experimental conditions reported in the worksheet (Table 1_SI). Then, after each UAE cycle, the extracts were centrifuged at 4000 rpm at 25 °C for 5 min, supernatants were collected after each extraction (n=3) in a falcon tube. The total extract was transferred in a round bottom flask and dried by a rotatory evaporator. The dried extract obtained for each sample was then solubilized in 1 mL of mobile phase and filtered by a nylon syringe filter (0.22 µm) before the analysis in the UHPLC-DAD system.

Exp No	Time	% water	Temperature	MK Yield %	LMK/AMK
**1**	10	1	25	76.8 ± 1.5	7.22 ± 1.76E-02
**2**	60	1	25	88.4 ± 2.2	6.75 ± 3.31E-02
**3**	10	100	25	43.9 ± 1.1	2.06 ± 3.13E-02
**4**	60	100	25	51.9 ± 1.0	1.79 ± 3.63E-03
**5**	10	1	80	92.1 ± 1.5	6.55 ± 9.28E-03
**6**	60	1	80	96.6 ± 0.8	6.31 ± 2.07E-02
**7**	10	100	80	85.9 ± 1.2	2.06 ± 8.57E-05
**8**	60	100	80	84.2 ± 2.7	0.97 ± 2.29E-03
**9**	10	1	50	92.4 ± 1.8	6.73 ± 1.32E-02
**10**	30	50	50	97.6 ± 2.0	6.00 ± 8.00E-02

Table S1: table reports ethanol and water mixture extraction experiments. The reported percentage of water is regarding the percentage of ethanol.

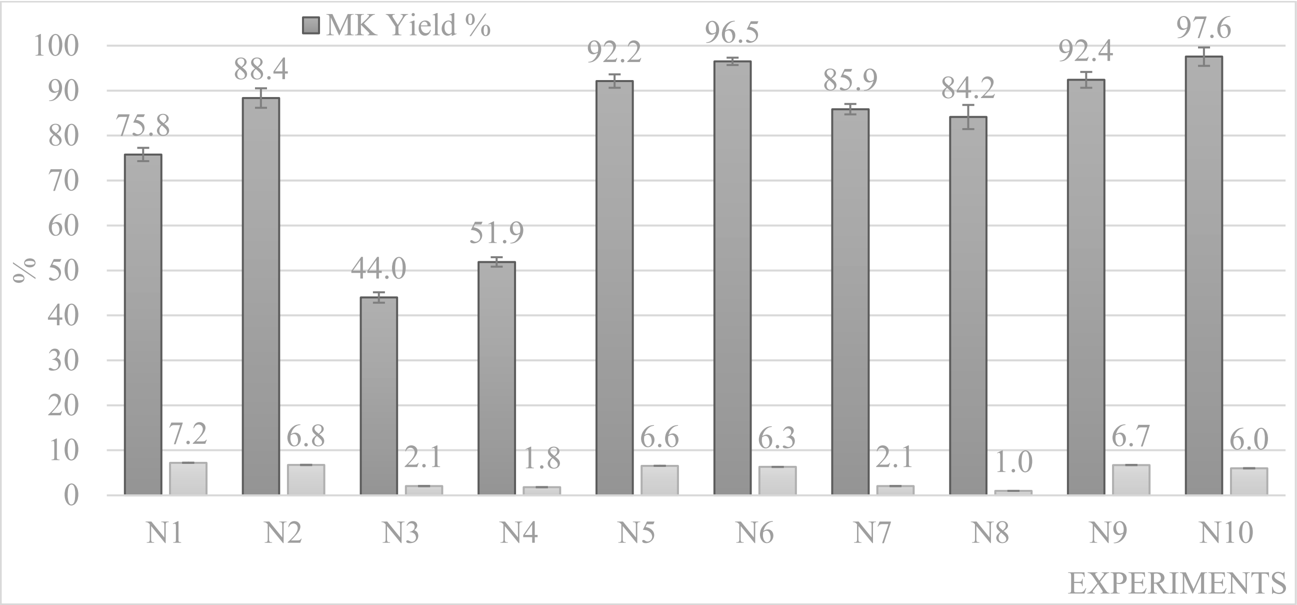

Figure S1: the graph reports the extractive condition results analyzed in terms of total yield of MK (dark grey) (%) and as ratio between the lactone and acid monacolin K forms (grey) (%).

2. Design of Experiment (DOE) for the optimization of UAE conditions for the extraction of LMK and AMK from RYR using a commercial mouthwash

To investigate the best UAE conditions of MK from RYR, by using as extraction phase a commercial mouthwash (composition reported in the material and method section), a DOE study was conducted. MODDE® Pro 13 Software (Sartorius Data Analytics) was exploited for this study. For DOE it was selected an optimization design, type D-optimal (3-level). The total extraction yield of MK, determined by UHPLC-DAD analysis, was selected as response to be maximized. Three main multilevel factors were considered: temperature, time and quantity of sample powder. Each factor was evaluated at three levels: temperatures= 25, 45, 80 ºC; time= 10, 30, 45 min; quantity of RYR sample powder =5, 50, 100 mg.

Furthermore, the worksheet describing the experiments to be performed was generated producing 15 experiments and 3 center points.

Experiment	Temperature °C	Time min	Powder mg	MK yield %	LMK/AMK

**N1**	25	10	5	42.2 ± 1.0	7.83 ± 3.95E-01
**N2**	80	10	5	75.6 ± 1,7	8.39 ± 7.69E-01
**N3**	25	45	5	62.5 ± 0.8	7.62 ± 2.35E-01
**N4**	80	45	5	86.6 ± 0.4	4.01 ± 7.70E-02
**N5**	45	30	5	83.2 ± 0.4	9.73 ± 5.29E-01
**N6**	25	10	100	24.2 ± 0.1	3.95 ± 2.95E-02
**N7**	80	10	100	76.0 ± 0.0	7.69 ± 0.00E+00
**N8**	80	10	100	75.3 ± 0.0	7.69 ± 0.00E+00
**N9**	25	45	100	29.6 ± 0.1	4.62 ± 6.28E-03
**N10**	80	45	100	80.6 ± 0.3	5.85 ± 3.55E-02
**N11**	45	30	100	38.7 ± 0.1	5.50 ± 8.69E-03
**N12**	45	10	50	40.5 ± 0.3	6.39 ± 1.14E-01
**N13**	45	45	50	64.7 ±1.1	7.55 ± 2.14E-01
**N14**	25	30	50	49.8 ± 0.1	6.35 ± 4.97E-02
**N15**	80	30	50	78.3 ± 1.2	6.35 ± 2.08E-02
**N16**	45	30	50	47.3 ± 0.6	6.71 ± 6.88E-03
**N17**	45	30	50	51.3 ± 0.1	6.54 ± 1.73E-01
**N18**	45	30	50	43.3 ± 0.4	6.67 ± 1.39E-01

Table S2: table reports the worksheet obtained with MODDE® describing the experiments to be performed and the related results.

3. Validation of the UHPLC-DAD method for the determination of LMK and AMK

3.1. Linearity and Sensitivity

The standard calibration curves for LMK and AMK were obtained by plotting the peak area of five incremental standard mix solutions versus the concentrations in the ranges 1.25-10.00 μg mL^{-1} and 0.50-8.00 μg mL^{-1} respectively. A satisfactory correlation coefficient ($R^2 > 0.999$) was obtained for the calibration curves of both analytes. Figure 2SI reports the obtained calibration curves for LMK and AMK.

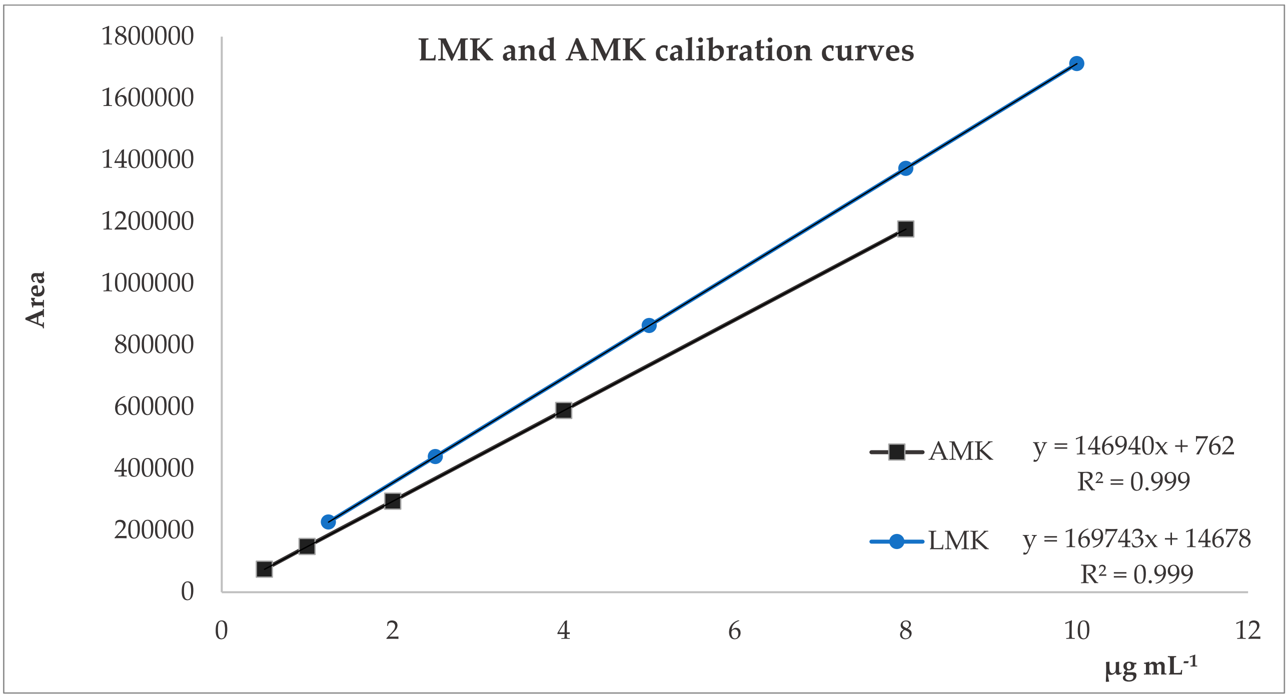

Figure S2: calibration curves of LMK and AMK

4. Development of UHPLC-DAD method to identify and quantify the chlorhexidine digluconate in the mouthwash extract

A new chromatographic method was developed to identify and quantify chlorhexidine digluconate (CD) in the mouthwash extract. The analyses were carried out by using the Jasco X-LC apparatus (Jasco Europe, Cremella, Italy) described in par. 2.3. The reversed phase column C4 (Kromasil 100, 50 x 3 mm, 2.5 µm) was equilibrated with a mobile phase consisting of acetonitrile and buffer solution 35:65 (v/v). Buffer solution was prepared using of 0.08 M sodium phosphate monobasic containing triethylamine (0.5%) and adjusted with 85% phosphoric acid to pH= 3.0. The analyses were performed under isocratic elution at a flow rate of 0.2 mL min^{-1} with total run-time of 4 min. The detection wavelength was set at 239 nm. The column temperature was kept at 25 °C. The fresh diluted (1: 1 x 10^5) CD standard aqueous solution (20%) was immediately injected. Then, the same solution was placed in the ultrasound bath for 45 min at 80°C (for 3 times). Then, the obtained solution was injected the chromatographic system.

The recovery of CD was evaluated comparing the peak area of CD before (CD1) and after (CD2) this thermic stress.

% Recovery CD: CD2 *100/ peak area CD1

Moreover, the eventually presence of the chromatographic peak of the degradation product p-chloroaniline was monitored.
